# Recommendations for Successful Development and Implementation of Digital Health Technology Tools

**DOI:** 10.2196/56747

**Published:** 2025-06-11

**Authors:** Rebecca Ting Jiin Loo, Francesco Nasta, Mirco Macchi, Anaïs Baudot, Frada Burstein, Riley Bove, Maike Greve, Holger Fröhlich, Sara Khalid, Arne Küderle, Susan L Moore, Valerie Storms, John Torous, Enrico Glaab

**Affiliations:** 1 Biomedical Data Science Group, Luxembourg Centre for Systems Biomedicine (LCSB), University of Luxembourg, 6 Avenue du Swing, Belvaux, L-4367, Luxembourg, 352 466644 ext 6186; 2 Marseille Medical Genetics (MMG), INSERM, Aix Marseille University, Marseille, France; 3 Department of Human-Centered Computing, Faculty of Information Technology, Monash University, Melbourne, Australia; 4 UCSF Weill Institute for Neurosciences, University of California San Francisco, San Francisco, CA, United States; 5 Department of Digitalization, Copenhagen Business School, Frederiksberg, Denmark; 6 Department of Bioinformatics, Fraunhofer Institute for Algorithms and Scientific Computing, Sankt Augustin, Germany; 7 Bonn-Aachen International Center for Information Technology (b-it), University of Bonn, Bonn, Germany; 8 Centre for Statistics in Medicine, Botnar Research Centre, University of Oxford, Oxford, United Kingdom; 9 Department of Artificial Intelligence in Biomedical Engineering (AIBE), Friedrich-Alexander-Universität Erlangen-Nürnberg, Bayern, Germany; 10 Department of Community and Behavioral Health, Colorado School of Public Health, Aurora, CO, United States; 11 Independent Patient Expert, PEP Matters, Hasselt, Belgium; 12 Department of Psychiatry, Beth Israel Deaconess Medical Center, Harvard Medical School, Boston, MA, United States

**Keywords:** digital health, health care technology, mobile health, telehealth, recommendations, user engagement, data privacy, regulatory compliance, interdisciplinary collaboration, user-centered design, data interoperability, technology, application, wearable, electronic health record, real-time, health monitoring

## Abstract

Digital health technology tools (DHTTs) have the potential to transform health care delivery by enabling new forms of participatory and personalized care that fit into patients’ daily lives. However, realizing this potential requires careful navigation of numerous challenges. This viewpoint presents the authors’ experiences and perspectives on the development and implementation of DHTTs, addressing both established practices and controversial topics. This article offers a practical guide organized into 10 recommendations derived from a multidisciplinary lecture series and associated workshop discussions on “Digital Health and Digital Biomarkers” held at the University of Luxembourg in 2023-2024. Key messages include the need to understand specific health care challenges, form interdisciplinary teams, incorporate patient feedback, select appropriate measurement technologies, ensure data integration and interoperability, apply advanced data science techniques, use scalable designs and open standards, comply with regulatory requirements, and maintain continuous evaluation and improvement. While the guide highlights essential practices, it also addresses contentious issues such as balancing innovation with regulatory compliance, addressing ethical concerns in artificial intelligence adoption, managing privacy versus the need for comprehensive data integration and open science, and managing the financial sustainability of DHTTs. The authors argue that digital health’s greatest potential lies in its ability to provide participatory and personalized care, but this requires a delicate balance between technological advances and ethical, legal, and social implications. Overall, this workshop-derived viewpoint aims to help health care professionals, engineers, developers, and researchers not only adopt best practices but also address and resolve the controversial aspects inherent in the development of DHTTs.

## Introduction

Digital health technology tools (DHTTs) have the potential to transform health care delivery by improving patient outcomes, increasing efficiency, and reducing costs. In the form of mobile health (mHealth) apps, wearable devices, telemedicine, molecular technologies, enhanced electronic health records, and associated advanced analytics, these tools are being used to optimize and streamline clinical workflows [1]. Entirely new forms of health care are envisioned that are not only more participatory and personalized but also seamlessly integrated into patients’ daily routines and environments. This seamless integration, ranging from real-time health monitoring to personalized treatment plans accessible from personal devices, could offer profound benefits, including improved access to care and patient engagement, timely interventions, and a more comprehensive approach to health management.

Despite promising benefits, DHTT development and implementation face several significant challenges. These include poor data interoperability with existing electronic health records [2], varying regional regulations [3], insufficient ethical oversight [4], and limited user-centered design [5]. Economic sustainability remains a critical barrier, with many technologies failing due to a lack of viable business models and funding strategies [6]. Additionally, most DHTTs lack thorough real-world validation beyond pilot studies, hindering their long-term adoption and improvement.

In this contribution, we aim to highlight essential practices for developing DHTTs and to address contentious issues in the field. These include the challenge of balancing rapid innovation with regulatory compliance, the need to address ethical concerns arising from artificial intelligence (AI) adoption in health care, the tension between maintaining patient privacy and achieving comprehensive data integration, and the struggle to ensure financial sustainability. By exploring these complex topics alongside suggesting best practices, we aim to provide a more comprehensive guide that acknowledges both the opportunities and challenges inherent in digital health innovation.

**Figure 1.**
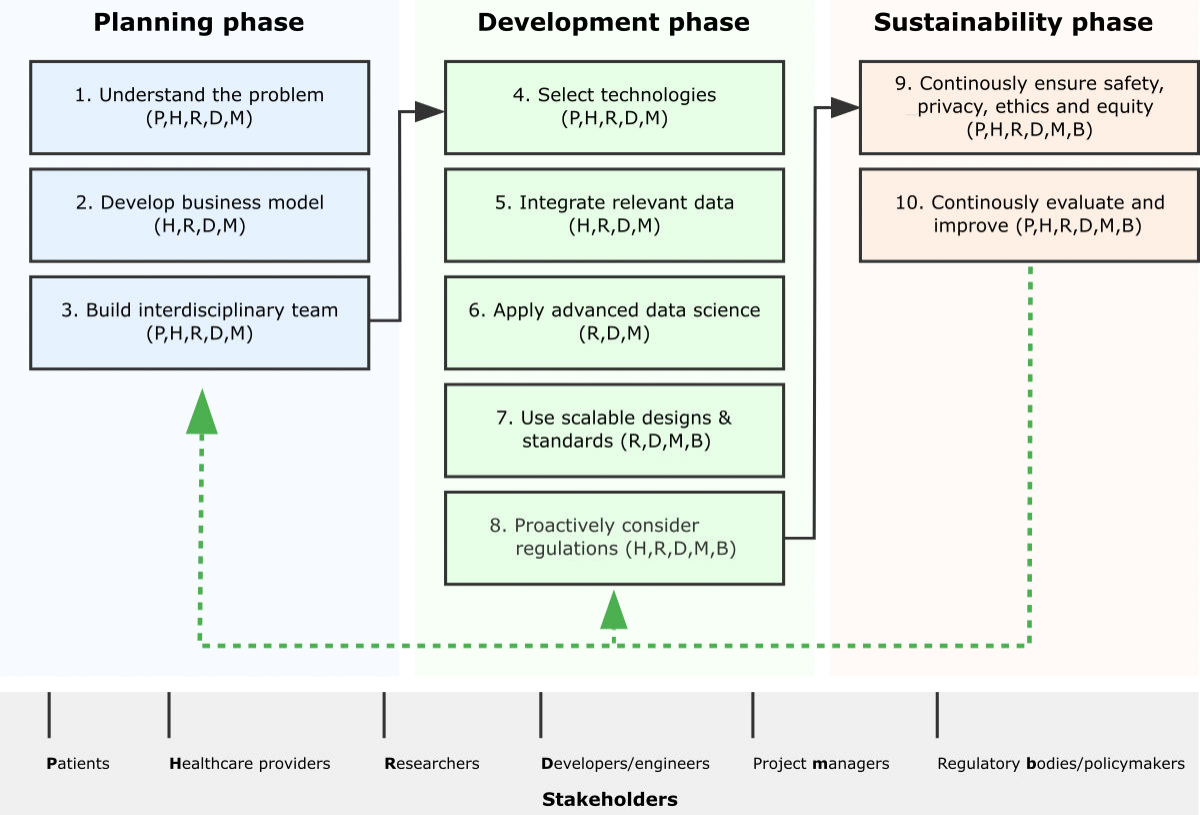
Generic workflow for the development of digital health technology tools (DHTTs). This figure illustrates common key steps in DHTT development, directly reflecting the 10 recommendations presented in this paper. The workflow is organized into 3 main phases: planning, development, and sustainability. Each numbered step corresponds to a specific recommendation and is accompanied by letter codes indicating the stakeholders directly involved in the project—P: patients and advocacy groups; H: health care providers; R: researchers; D: developers and engineers; M: project managers; B: regulatory bodies and policymakers. The workflow progresses from left to right, with arrows indicating the primary flow between phases. The green dashed arrow represents the continuous evaluation and improvement process (recommendation 10) that spans all phases, emphasizing the iterative nature of DHTT development. We note that this figure presents a simplified, generic workflow, and specific DHTT projects may often have additional subphases or phases beyond this basic scheme, depending on the requirements, complexity, and scope of the digital health solution being developed. The actual workflow may be more complex and iterative, with potential variations for different types of DHTTs and organizational contexts.

We target a broad readership spanning health care professionals, engineers, developers, and researchers to provide practical guidance and step-by-step recommendations for common pitfalls and optimize key aspects of DHTT design. The focus is not on pure digital technology/hardware implementation for the health care sector but on DHTT development in general, including mobile apps and data analytics that build on existing hardware. While not aiming to present exhaustive or project-specific methodologies, our proposed recommendations highlight important generic considerations at each development phase based on lessons learned from real-world studies, from initial scoping and planning through launch and to full sustainability (see also the schematic overview in Figure 1). The development of these recommendations stems from insights gathered during a lecture series on “Digital Health and Digital Biomarkers” at the University of Luxembourg. This event brought together experts from health care, engineering, technology, and ethics. Specifically, our recommendations were developed through a series of workshops and collaborative discussions following the lecture series in 2023-2024, where participants collectively proposed and refined suggestions until reaching a consensus. All recommendations were adopted unanimously through discussion, making planned formal voting procedures unnecessary. The discussions revealed the significant potential and challenges of DHTTs, underscoring the necessity for a practical guide to navigate these complexities effectively. Core themes identified include the need for human-centered design, rigorous evaluation, ethical oversight, and continuous quality improvement. While our guide focuses on opportunities, each recommendation also highlights common risks to avoid or controversial topics that are subject to debate. In addition, we point out that these recommendations apply with varying relevance across five main types of DHTTs: (1) hardware-focused solutions like wearables and medical devices, (2) software-focused tools such as mobile apps and AI algorithms, (3) integrated hardware-software systems, (4) clinical decision support systems, and (5) consumer-focused health apps. Each type faces distinct combinations of technical, regulatory, and implementation challenges, requiring careful prioritization of development principles. Insights from both successful implementations and notable setbacks in the field are presented. Our goal is to provide readers with strategies to avoid common pitfalls and develop effective digital capabilities that improve health care. By learning from experiences, developers can avoid repeating known mistakes, build on established best practices, and ultimately create more impactful and sustainable DHTTs.

## Recommendation 1: Understand the Problem to Solve

The first step in developing DHTTs is to fully understand the specific health care challenge and associated knowledge gaps, with a focus on the patients’ and caregivers’ needs. Relevant aspects to consider include specifying the target population, research questions, and required data collection. Also important are potential predictive capabilities, intervention optimization, and anticipated impact on health care and patient outcomes. Clearly defining the scope of the problem and the goals to be achieved is essential. Involving the end beneficiary from the start lays the foundation for an effective, user-centered solution.

User-centered design is an approach that prioritizes the needs, preferences, and limitations of end users throughout the design process. In DHTT development, this means involving patients, health care providers, and other stakeholders from the initial concept through implementation. Despite its benefits, user-centered design is not always adopted due to factors such as time and resource constraints, technical bias, the assumption of expertise by health care professionals or technologists, and an overemphasis on meeting regulatory requirements. Commonly used alternative practices include technology-driven design, which focuses on technical capabilities rather than usability; expert-driven design, which relies solely on professional opinion without user input; market-driven design, which prioritizes marketable features; and regulatory-driven design, which focuses primarily on compliance. We believe that these approaches run the risk of creating solutions that are difficult to use, do not address real user needs, have low adoption rates, or are ineffective in real-world environments despite being compliant. In contrast, user-centered design helps mitigate these risks by ensuring that DHTT solutions are not only technically sound and compliant but also truly useful, usable, and responsive to real user needs. This approach, while potentially more time- and resource-intensive initially, can lead to more effective and widely adopted DHTTs in the long run. A successful example is the study by Giunti et al [7] on multiple sclerosis patients, where a thorough exploration of user needs led to an effective gamified mHealth app for fatigue management, demonstrating the value of early problem understanding in DHTT development.

In addition to considering the specific health care challenges, it is essential to address the economic sustainability of the DHTTs. This involves evaluating the “willingness to pay” or establishing a viable business model. Understanding who will finance the solution once it is ready for implementation is critical, as many digital health technologies fail to be adopted due to a lack of financial backing. This financial assessment should take into account various potential payers, including health care systems, insurance companies, patients, or other stakeholders, and consider the overall value proposition of the solution in the context of the health care market.

To identify potential areas for improvement, key epidemiological factors, disease mechanisms, and current standards of care, the clinical literature should be thoroughly reviewed. In addition, relevant systematic reviews and meta-analyses should be studied to synthesize current evidence on disease burden, risk factors, prognosis, and existing interventions. Importantly, to contextualize the results of these systematic reviews, consultation with patients, caregivers, and medical experts provides essential insight into real-world needs and challenges that must be addressed [8]. The Patient Focused Medicines Development initiative has published a dedicated “Patient Engagement Quality Guidance” [9] for this purpose.

Moreover, professional societies’ clinical practice guidelines can offer valuable insights into recommended strategies for the diagnosis, screening, treatment, and ongoing monitoring of various conditions [10]. To assess how a new DHTT differs from or improves upon existing solutions, the competitive landscape should be analyzed. For this purpose, the World Health Organization’s digital health strategy [11] provides insights into international trends and developments in the field. This strategy specifically emphasizes integrating digital interventions into national health care systems, requiring evidence-based, equitable solutions with strong governance and sustainable financing while prioritizing interoperability and standards. A comprehensive approach considering the broader health care ecosystem, national priorities, and international collaboration is recommended for effective digital health implementation. In summary, the success of DHTTs hinges on their ability to effectively address the specific clinical issues identified. This requires a deep understanding of the disease burden, risk factors, prognosis, current interventions, and the patient’s clinical journey from initial symptoms and diagnosis through different treatment stages and long-term management. Such knowledge should be gained from systematic reviews, meta-analyses, and direct consultations with patients, caregivers, and medical professionals to ensure alignment with real-world needs.

## Recommendation 2: Develop a Viable and Sustainable Business Model

Developing a viable and sustainable business model is essential to the success and durability of DHTTs that have established a strong evidence base [12]. This aspect is often overlooked or underestimated, leading to the failure of many promising DHTTs, regardless of their technical merits or user-centered design. A well-designed business model is essential to attract investment, ensure long-term sustainability, and ultimately deliver value to users and the health care system. For example, Proteus Digital Health’s Food and Drug Administration (FDA)–approved smart pill system [13] failed despite US $500M in funding, as its business model could not justify high costs to insurers. This case underscores why DHTT developers must not only create innovative technologies but also develop viable business models aligned with health care system economics.

An overview of key considerations for developing a viable DHTT business model is provided in Textbox 1. For detailed guidance on user-centered design and regulatory compliance, which are important complementary aspects of DHTT development but distinct from core business model considerations, see Recommendations 1 and 8, respectively.

**Textbox 1. Essential components of a sustainable DHTT business model encompassing financial, strategic, and market considerations, among others.**

- Value proposition: clearly define the unique value proposition, aligning it with identified user needs and the broader health care ecosystem.
- Revenue streams: identify multiple potential revenue streams, such as direct user payments, subscription models, licensing to health care providers, partnerships with insurance companies, or ethical data monetization [14].
- Cost structure: understand and optimize the cost structure, including development, maintenance, user acquisition, and scaling costs.
- Reimbursement pathways: for clinical solutions, explore potential reimbursement pathways, working with insurance companies and understanding health care policy trends.
- Strategic partnerships: identify partnerships that can provide access to users, enhance credibility, or offer additional revenue streams.
- Intellectual property (IP): conduct a thorough IP landscape analysis and develop a robust IP strategy. This helps maintain a competitive advantage and can attract investment.
- Scalability requirements: consider technical scalability needs (eg, cloud infrastructure and load balancing) and associated costs as the user base grows.
- Defensibility: evaluate how your technology can be protected against potential infringement or replication. This might involve technical measures, legal protections, or continuous innovation.
- Market and competitor analysis: perform comprehensive market research to understand the current landscape, identify unmet needs, and analyze both direct and indirect competitors. Use these insights to position your DHTT effectively.
- Return on investment [ROI] metrics: define clear ROI metrics that align with the value proposition and needs of the potential investors. These should include not only financial measures but also health outcomes and system efficiencies.

## Recommendation 3: Build an Interdisciplinary Network and Incorporate Feedback

**Figure 2.**
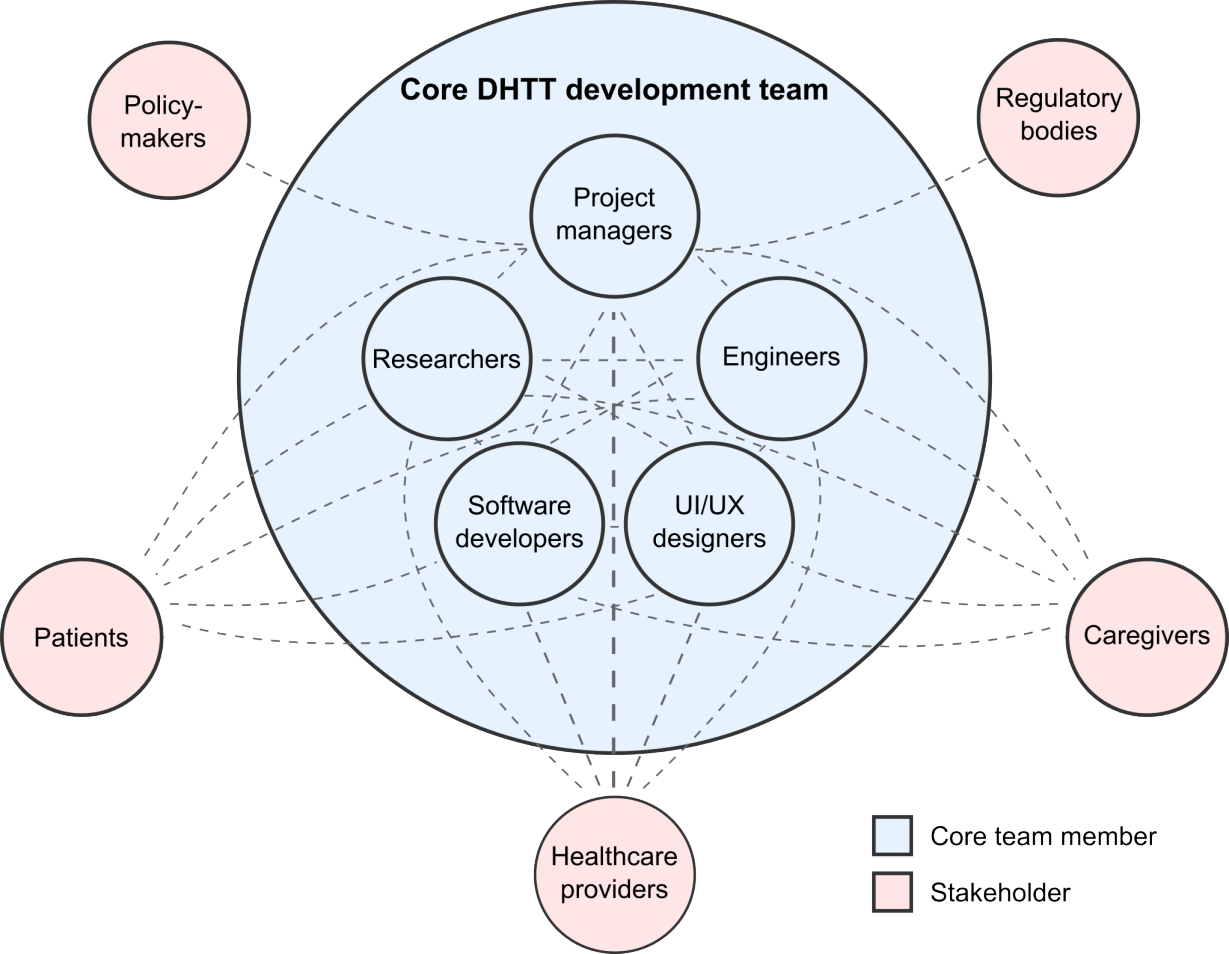
Interdisciplinary team structure and direct stakeholder involvement for digital health technology tool (DHTT) development. This figure illustrates a typical composition of an interdisciplinary team for DHTT development, as outlined in recommendation 3. Interactions are highlighted by the gray dotted lines. The core development team (blue) consists of project managers, researchers, engineers, software developers, and user interface and user experience (UI/UX) designers. Key stakeholders who are directly involved in the project design (shown in pink), including patients, caregivers, health care providers, policymakers, and regulatory bodies, are shown interacting with the core team. These interactions emphasize the importance of diverse perspectives and collaborative input throughout the DHTT development process.

Successful development and implementation of digital health approaches benefit from extensive interdisciplinary collaboration through both core team members and external network partners. The core development team typically includes key internal stakeholders such as researchers, engineers, software developers, and user interface and user experience designers, while a broader network of essential collaborators encompasses patients, caregivers, health care providers (eg, doctors and nurses), regulatory bodies, and policymakers (see Figure 2). The value of this networked approach is illustrated by an mHealth app for chronic obstructive pulmonary disease (COPD) monitoring [14]. The development team coordinated with physicians, nurses, and patients to create an app that balanced clinical needs with a user-friendly design. This approach resulted in high user satisfaction and improved COPD management, showing how interdisciplinary collaboration leads to effective DHTTs.

Bridging the gap from research to practical applications requires integrating expertise from academic, industrial, clinical, ethical, legal, and regulatory domains. Additional stakeholders such as funding organizations, advocacy groups, and IT suppliers [15] contribute through resources, advocacy, and infrastructure support. These networks facilitate technology development through consultation, testing, and deployment, with different engagement approaches needed for end users (eg, via participatory design and usability studies) versus technical stakeholders (eg, through workshops and advisory panels).

Ideally, all relevant stakeholders should be involved from the earliest stages and throughout the project lifecycle. Early engagement helps understand needs and constraints, essential for design and successful implementation. In cases of limited experience with digital health technologies, providing education and training to stakeholders may be advisable. Partnerships should be formalized with clear roles and responsibilities. Diverse viewpoints can be incorporated through participatory design sessions. Effective communication channels and decision-making guidelines should be established to maintain stakeholder engagement and trust. An advisory board can provide continuous feedback and support.

Throughout the lifecycle of digital health technologies, it is important to engage end users and collect feedback. This includes predeployment activities such as participatory design and usability testing, as well as postdeployment methods such as satisfaction surveys and analysis of usage data. Various research methods can be used to understand patients’ priorities, values, concerns, and preferences. Codeveloping applications with patients and providers through iterative prototyping can yield new insights. For this purpose, inclusive research design methodologies can provide useful strategies on how to address the dynamic relationship between DHTT developers and external stakeholders [16,17]. Key approaches include Participatory Action Research, which involves stakeholders as co-researchers; community-based participatory research, emphasizing equitable partnerships with community members; co-design, involving end users from the start; and inclusive user experience research, ensuring diverse user representation. These methodologies are particularly beneficial in DHTT development as they lead to solutions that are more relevant, usable, and equitable [18,19]. By actively involving end users and diverse stakeholders throughout the research and development process, these approaches increase the likelihood of creating DHTTs that effectively meet user needs and achieve successful adoption and implementation.

User feedback reveals both opportunities and challenges in DHTT implementation. For instance, understanding patient struggles with diabetes management led to successful automated insulin delivery systems [20], while concerns about privacy, complexity, and workflow disruption can hinder adoption [21,22]. This skepticism can be particularly pronounced with advanced AI-based solutions, especially in areas where patient-related outcomes are critical [23]. Addressing this requires transparency in AI decision-making, clear explanations of the evidence base, clinician involvement, and emphasis on augmenting rather than replacing clinical judgment. By proactively considering concerns and engaging users throughout the development process, developers can create solutions that are more likely to be adopted and provide real value in clinical practice.

## Recommendation 4: Select Appropriate Measurement Technologies for Biomarker Data Collection

This recommendation focuses on selecting technologies for collecting biomarker data in DHTTs. Biomarkers in this context refer to objectively measured indicators of biological processes, pathogenic processes, or responses to therapeutic interventions that can be captured by digital technologies. Biomarker measurement approaches used within a DHTT should be carefully evaluated for clinical suitability and technical soundness. They must also be easy to use, inclusive, and either already approved by regulatory authorities or have a realistic pathway to approval for the intended use. In this context, new digital sensor technologies, such as those for assessing heart rate variability, gait characteristics, sleep patterns, or activity levels, can play critical roles in monitoring and predicting health outcomes. These sensors can provide continuous, objective, and sensitive indicators of a person’s medical status, enabling more accurate diagnostic and prognostic analyses and faster and more patient-tailored responses [24]. However, the capabilities, limitations, and evidence base should be considered when identifying technologies and data types that are sufficiently accurate and specific for the health care challenges being addressed. Relevant factors such as sensitivity, specificity, reproducibility, and clinical validity should first be critically appraised through rapid and rigorous evaluation, with results ideally documented through published studies. For example, a study of 7 wrist-worn devices [25] found good heart rate accuracy but poor energy expenditure estimates (27.4% to 92.6% error), highlighting the importance of rigorous validation across diverse populations before clinical implementation.

Apart from validating measurement accuracy for all digital measurement devices, sampling frequency and duration should capture relevant physiological dynamics. Apart from assessing hardware technology, analytical methods for the extraction of digital biomarkers and endpoints should also be validated. In addition, project participants must ensure that the technologies used to collect, analyze, and present data for digital health applications are appropriate for the target population, which may have special needs related to disabilities or digital literacy. Moreover, it is imperative to address two distinct sources of bias that may arise in digital health applications. First, data bias occurs when the collected data does not accurately represent the diverse populations affected by the health issues in question, often due to sampling methods or historical health care access disparities. Second, algorithmic bias can arise independently through technical design choices in the modeling process, such as feature selection, model architecture, or optimization criteria, which may lead to varying performance across different patient subgroups even with representative training data. Both types of bias require distinct mitigation strategies: improving data collection diversity and representation for the former, and careful algorithm design and validation across different populations for the latter.

Finally, pilot testing under real-world conditions with diverse populations helps refine the technology and evaluate generalizability [26], particularly for wearable devices where user engagement is essential. Research shows this participatory approach fosters long-term user motivation [27].

## Recommendation 5: Integrate Relevant Data and Ensure Interoperability

The integration of digital health data across different systems and modalities remains a major challenge in health care. While comprehensive assessment of symptoms through multiple data sources—such as digital outcome measures, patient-reported outcomes, electronic health records, and (multi-)omics data—could provide a more complete view of patient health status [28,29], careful evaluation of the cost-benefit ratio for each additional data source is essential. In many cases, a focused selection of one or two key modalities may be sufficient to achieve the intended health care outcomes, particularly given that the current reality often limits data collection due to interoperability barriers and integration costs. However, the benefits of even basic data integration and interoperability are illustrated by a study of health information exchange implementation in New York [30], where simply enabling emergency department physicians to access basic patient information across providers led to 30% lower hospital admission rates and significant cost savings.

To ensure interoperability, standard ontologies, semantics, and data models should be adapted [2,31], and Fast Healthcare Interoperability Resource (FHIR) interfaces [32] can be used for seamless data exchange across applications and systems. Clinical quality standards and common data elements [33,34] can also guide multimodal integration, while standardized software pipelines [35] and reference datasets support robust data processing. Furthermore, data management should follow the FAIR (Findability, Accessibility, Interoperability, and Reusability) principles [36], ensuring the data is findable (unique identifiers, rich metadata), accessible (standardized retrieval protocols), interoperable (shared knowledge representation), and reusable (clear licensing and provenance). This increases the value of digital health data for research, clinical decision-making, and the development of new DHTTs.

## Recommendation 6: Apply Advanced Data Science Techniques Where Appropriate

Data science approaches in health care range from simple visualization and knowledge-based systems to complex AI models. While advanced AI methods can analyze diverse digital health data to generate highly accurate prediction models [37], simpler interpretable approaches can be equally valuable. For example, the heart failure diagnosis system by Choi et al [36] combined expert rules with machine learning (ML), achieving 98% concordance with specialists while maintaining interpretability.

The choice between simple and complex techniques should prioritize clinical needs, with interpretable methods, for example, using compact decision rule sets [38] or basic visualization tools, preferred when explainability is important for clinical adoption and achievable without significant loss of accuracy. Alternatively, hybrid solutions that combine interpretable modeling with more sophisticated algorithms can provide both transparency and high performance. Whenever possible, prior domain knowledge should guide the selection of analytical methods.

Importantly, the constraints inherent in both data sources and modeling approaches must be considered. For predictive models, the performance should be carefully evaluated with a focus on both discrimination and calibration capabilities. This evaluation should use clinically relevant performance metrics, complemented by robust train-test methodologies such as cross-validation and bootstrapping [39]. In the medical domain, it is particularly important to address common pitfalls in train/test splits, such as not splitting data at the participant level when multiple data points per participant are present, or not adequately handling class imbalances [40]. These oversights can lead to misleading performance estimates and biased models. Models rely heavily on training data, which often contain biases, and therefore robust evaluation and independent validation using datasets with information from diverse real-world populations are essential. In addition, model safety, robustness, and fairness [41] should be evaluated through rigorous independent testing. This can include measuring model performance consistency across different patient subgroups, assessing model interpretability through techniques like feature importance analysis, stress testing with out-of-distribution inputs, and systematic evaluation of error rates and their clinical implications using standardized benchmarking datasets [42,43]. Best practices for reproducible ML research should also be followed [44].

Overall, by combining validated data science and integration techniques with clinical expertise, actionable and trustworthy insights can be extracted from digital health data.

## Recommendation 7: Use Open Standards and Open Science Approaches

When developing DHTTs, researchers often face a tension between focusing on scientific objectives—such as proof-of-concept validation and publication—versus meeting industrial-grade development standards. While academic prototypes may initially prioritize demonstrating scientific validity, their eventual translation to clinical practice requires consideration of industrial standards. This transition often involves significant refactoring of code and careful evaluation of open-source license compatibility for commercial use.

As DHTTs move toward clinical implementation, developers should increasingly prioritize open standards to ensure long-term viability and interoperability. While initial research prototypes may use simpler architectures, production-ready systems need to consider standard approaches for deployment and integration [45]. Integration with other systems is also facilitated by adopting open science principles in digital health development. Openly sharing codes and models promotes transparency, reproducibility, distribution, and widespread adoption of DHTTs. Using dedicated platforms such as GitHub and GitLab for code sharing and repositories such as Kipoi [46] for trained models can significantly contribute to the open science ecosystem. These practices not only enhance peer collaboration and innovation but also build trust and credibility within the user community.

However, while we advocate for open science principles in DHTT development, we recognize the need to balance these with patient privacy concerns and viable business models, especially for commercial entities. To reconcile these potentially conflicting needs, we propose the following strategies: (1) implementing tiered access models in which basic functionality is open source while advanced functionality remains proprietary, (2) adopting an open core model with proprietary add-ons, (3) using time-shifted releases to maintain a temporary market advantage, (4) engaging in collaborative research partnerships involving both open and proprietary research aspects, (5) contributing to data commons while maintaining proprietary analytic tools, (6) publishing standards openly while keeping specific implementations proprietary, (7) using patent and open source dual licensing (ie, protecting core innovations through patents while offering open-source licenses for noncommercial or research use), and (8) implementing privacy-preserving data sharing approaches (see Recommendation 9).

These approaches allow DHTT developers to contribute to the open science ecosystem and encourage innovation and transparency while maintaining competitive advantages and sustainable business models. The key is to strategically decide which components to open and which to keep proprietary based on business goals and market dynamics. By adopting these strategies, developers can strike a balance between open science principles and commercial viability, potentially leading to more robust and widely adopted DHTTs.

Finally, developers should adopt published standards and terminologies (IEEE 11073 [47], FHIR [48], CDISC [49]) to enable platform-agnostic data integration and sharing. By building on stable, well-maintained tools and foundational technologies, software tools are more likely to remain relevant and maintainable.

In summary, the adoption of open standards and open science approaches can help to ensure that DHTTs are interoperable, reusable, and compatible with other medical devices and health care information systems.

## Recommendation 8: Proactively Consider Regulations and Align With Health Care Goals

While regulatory compliance is a legal obligation for DHTT developers, proactive consideration of regulatory requirements and health care goals from the early stages of development can lead to more efficient and effective solutions. Relevant laws and regulations in target markets should be identified upfront and proactively considered in the early stages of project design. Throughout the development of the DHTT, compliance with these requirements must be monitored, especially when major changes are made to the functionality or applications of the technology.

Early engagement with regulatory frameworks and authorities, such as the FDA and the European Medicines Agency, allows developers to incorporate compliance requirements into the initial design, align with health care goals, and adapt to future changes while avoiding costly late-stage modifications. In particular, when handling health information, compliance with data protection regulations (HIPAA [Health Insurance Portability and Accountability Act] in the United States and GDPR [General Data Protection Regulation] in the European Union) [50,51] is essential, requiring technical controls such as encryption and secure storage [52]. Information protection standards compliance can, for example, be demonstrated through certifications such as HITRUST (Health Information Trust Alliance). The emerging European Health Data Space framework will further standardize data exchange across European Union countries.

Beyond data protection, adopting trustworthy AI practices in accordance with recommendations from authoritative organizations such as the FDA and the Organisation for Economic Co-operation and Development (OECD) will also help ensure that ethical standards are upheld. Important resources include the FDA guidance on predetermined change control plans for AI/ML-enabled devices [53] and on digital health technologies for remote data acquisition in clinical investigations [50], as well as the OECD’s “Recommendation on Health Data Governance” [51] and “AI Principles” [54]. Additionally, the Digital Health Policy Navigator software by the FDA supports developers of digital health technologies in understanding the regulatory landscape [55]. For the European Union, the recent introduction of the AI Act [56] marks a significant step toward standardizing AI practices, focusing on risk assessment and compliance for AI systems used within the European Union. However, as there are currently still many regulatory gaps in determining the compliance of DHTTs with ethical, safety, and efficacy norms in many countries, various alternatives to regulatory agency-based assessments have been proposed to bridge these gaps. Examples include using information from prior reimbursement decisions or DHTT evaluations by large hospital networks [3].

In parallel, aligning DHTTs with established national and international health care goals enhances their relevance and impact. Frameworks such as the Institute for Healthcare Improvement’s Triple Aim [57], focusing on patient care experience, population health, and cost reduction, and the U.S. Institute of Medicine’s “Six Domains of Healthcare Quality” [58] should be taken into account. Finally, for developers targeting the European market, considering initiatives such as the EU4Health Programme can help to align the solution with the proposed visions for a healthier European Union.

## Recommendation 9: Ensure Safety, Privacy, Ethics, and Equity

**Figure 3.**
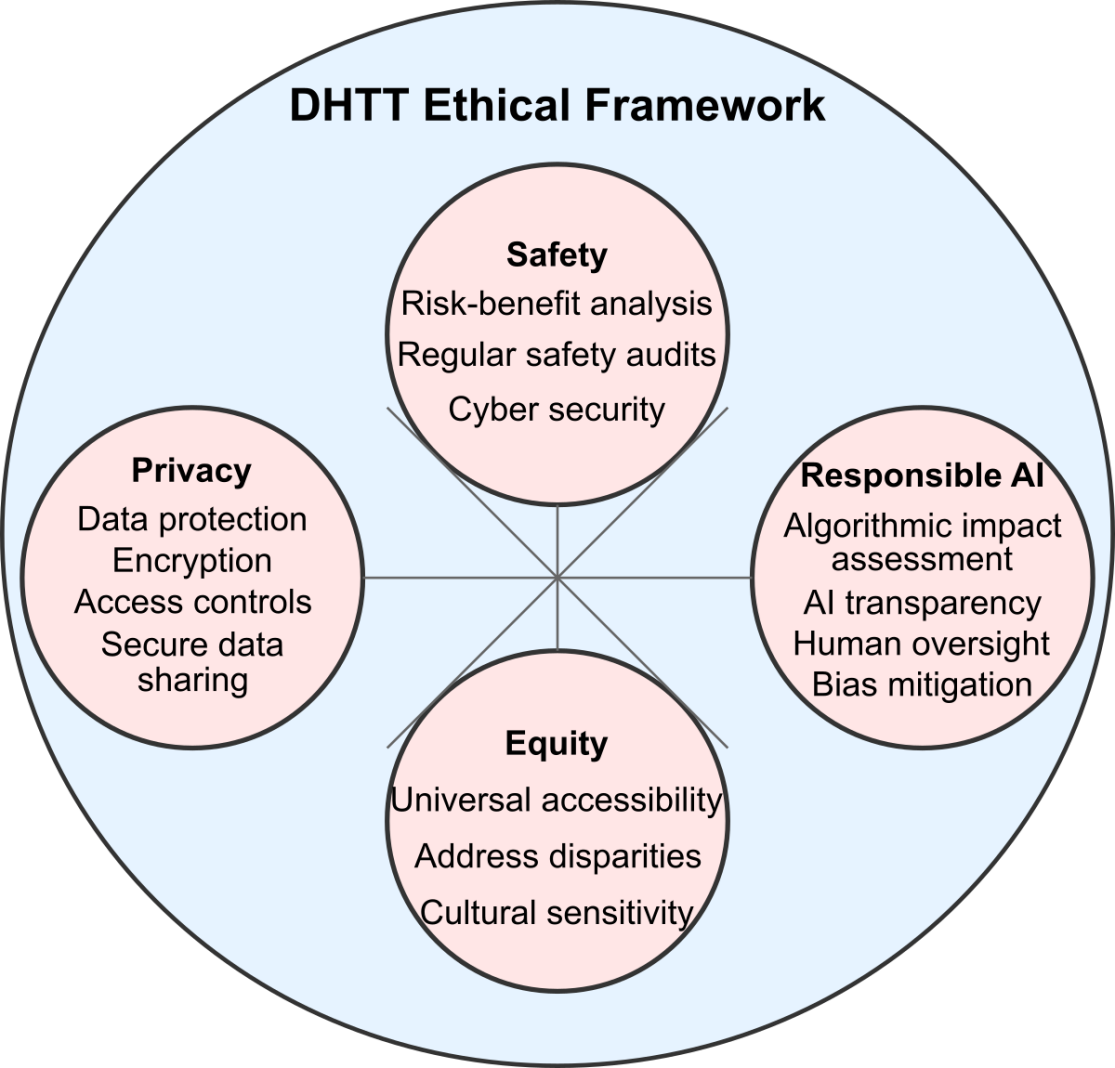
Digital health technology tool (DHTT) ethical framework. Four interconnected pillars—safety, privacy, responsible artificial intelligence (AI), and equity—provide critical considerations for ethical DHTT development, including risk-benefit analysis, data protection, algorithmic assessment, and health care disparity measures.

DHTT development requires integrating ethics, equity, safety, privacy, and responsible AI practices (Figure 3), with careful risk-benefit analysis to prevent potential harms, such as overreliance on treatment. Already in 2023, we have seen several cases of harm caused by mental health large language models that were deployed in manners not consistent with ethical guidelines or safety procedures in place [4]. It is also essential to protect patient privacy, for example, through deidentification, encryption, secure and private data sharing, access controls, and consent processes [59]. Differential privacy, secure multiparty computation, federated learning techniques, and privacy-preserving synthetic data generation [60,61] can minimize data exposure risks while maintaining data utility for research and development. If data transfer and communication between different devices are needed, best practices for information and cybersecurity, such as ISO/IEC 27001 [62], the NIST (U.S. National Institute of Standards and Technology) Cybersecurity Framework [63], and Cloud Security Alliance’s Cloud Controls Matrix [64], should be implemented. Beyond legal requirements, the inclusive, culturally sensitive design of DHTTs will promote usability across diverse user populations. To this end, potential disparities in access, technology adoption, and health impact across population subgroups should be actively addressed. Health equity and justice can be promoted by making digital health innovations accessible and affordable to underserved communities [65]. Moreover, AI-based systems must maintain human oversight, empowering clinicians to understand and override recommendations to ensure AI augments rather than replaces clinical judgment. Finally, developers need to adopt responsible AI practices such as bias detection, algorithmic impact assessments, and transparency to evaluate risks and mitigate biases [66], and there should be human oversight of any automated decisions [67].

## Recommendation 10: Continuously Evaluate and Improve

Throughout the life span of DHTTs, continuous monitoring, assessment, and iterative improvement are required to ensure accurate and reliable results, security, and functionality. Although DHTTs that qualify as medical devices require clinical evaluation for certification, for DHTTs in general, including noncertified mHealth apps, the rigor of evaluations can vary significantly [68]. While some undergo comprehensive assessment through randomized controlled trials (RCTs), such as the web-mediated follow-up algorithm for lung cancer patients evaluated by Denis et al [69], many mHealth apps focus only on usability rather than validated clinical outcomes. Larsen et al [70] found that among 73 top-ranked mental health apps, only 2 provided primary evidence, 1 cited published literature, and 14% described development with lived experience. None referenced certification processes. However, the importance of rigorous evaluation has already been demonstrated in practice. In the MONARCA II trial, a smartphone-based monitoring system for bipolar disorder showed promise in pilot studies but failed to demonstrate significant benefits in reducing symptoms when subjected to a full RCT [71]. Increasing the number of DHTTs subjected to high-quality clinical evaluations, including RCTs where appropriate, would further strengthen the evidence base for their use in clinical practice, potentially expedite both regulatory approval and insurance reimbursement processes, and help identify ineffective solutions before widespread adoption. Rigorous validation must occur already prior to deployment [26].

Once a digital health application is implemented, key performance metrics should be tracked across several domains. Technical performance measures (eg, system availability and data completeness rates) help ensure reliable operation, while clinical impact metrics (eg, diagnostic accuracy and workflow completion times) assess health care delivery improvements. User engagement tracking (eg, retention rates and feature adoption) provides insights into application acceptance and utility, and safety and quality measures (eg, error rates and security incidents) monitor system reliability and risk management. These metrics should be regularly monitored to identify potential problems and ensure the application continues to meet its intended goals.

Validated frameworks for continuous data monitoring and evaluation tasks, such as periodic auditing and automated performance tracking, should be adopted, for example, using open-source tools such as Prometheus and Grafana [72,73]. In addition, periodic reassessment of the solution against evolving best practices and regulations will help ensure continued relevance. Furthermore, postmarket surveillance, including proactive risk identification, is essential for patient safety [74]. This should be complemented by the establishment of strong data governance processes to maintain the quality and integrity of data inputs and algorithmic models over time. It is also advisable to implement control procedures that can quickly identify and address unintended consequences or privacy risks arising from the use of the digital application [75]. Finally, regular software updates and enhancements can further improve the functionality and clinical value of the digital solution. By focusing on these aspects of postdeployment evaluation and improvement, developers can ensure their DHTT remains effective, safe, and relevant in an evolving health care landscape.

## Discussion

### Implementation Considerations and Real-World Applications

Integrating digital technologies into health care presents both opportunities and significant challenges. In this guide, we have provided recommendations for implementing effective DHTTs based on real-world experience and evidence-based best practices.

Our recommendations cover a wide range of issues relevant to the entire process, from initial DHTT development in research settings to clinical translation. While we have sought to provide generically applicable guidance, we recognize the different requirements between clinical DHTT development and emerging technologies, which often require preliminary research efforts. This distinction is particularly relevant in the early stages of academic digital health projects. During conceptualization and pilot phases, research-based approaches such as systematic reviews are common, while practical implementation and deployment strategies are considered in later operational development phases. We acknowledge these distinctions, emphasizing that the path from research to operational development may vary depending on the nature and maturity of the DHTTs.

As outlined in the Introduction section, we identified 5 main types of DHTTs, each requiring a different emphasis on specific recommendations. For hardware-focused solutions (type 1), Recommendations 4 and 5 (measurement technologies and data integration) are most important, while software-focused tools (type 2) benefit most from Recommendations 6 and 7 (data science and scalable design). Integrated hardware-software systems (type 3) require a strong emphasis on Recommendations 2 and 3 (business model and interdisciplinary teams). Clinical decision support systems (type 4) should prioritize Recommendations 1, 6, and 8 (problem understanding, data science, and regulations), while consumer health applications (type 5) need to emphasize Recommendations 1, 3, and 9 (problem understanding, user feedback, and ethics). However, all recommendations retain some degree of relevance across the DHTT spectrum due to the interconnected nature of digital health technologies.

Several DHTT development studies have already demonstrated the practical application of our recommendations. For example, the integration of wearables into health systems by Kaiser Permanente and Ochsner Health System addresses technology selection and data security [76] (Recommendations 4 and 9). These systems effectively used wearables to monitor vital health parameters and integrated this data into electronic health records to improve clinician-patient communication. Similarly, a study to monitor hospitalized COVID-19 patients using ML with wearable technology successfully applied appropriate technology selection (Recommendation 4) and advanced data science approaches (Recommendation 6) to improve patient monitoring and predictive health care [77].

These studies demonstrate that carefully implemented and continuously evaluated digital health approaches can successfully transform health care delivery when following structured development guidelines.

### Conclusions

In this paper, we have proposed a set of recommendations aimed at addressing key considerations in the lifecycle of digital health technologies and tools (DHTTs), from initial planning to long-term maintenance. These guidelines, based on a combination of literature review and expert consensus, provide a framework for approaching the complex task of integrating digital technologies into health systems.

While not exhaustive, these recommendations provide a starting point for health care professionals, developers, and researchers working on DHTTs. They cover common practical issues such as problem definition, interdisciplinary collaboration, user engagement, technology selection, data integration, advanced analytics, scalability, regulatory compliance, and ethical considerations.

By considering these guidelines, teams may be better equipped to meet the challenges of creating effective, user-centered, and ethically sound digital health solutions. We believe that key success factors include adopting human-centered design principles, conducting rigorous evaluations, maintaining ethical oversight, and committing to continuous improvement based on user feedback and new evidence.

However, it is important to recognize that these recommendations represent a foundation rather than a comprehensive solution. The rapidly evolving nature of both health needs and digital technologies requires ongoing refinement of best practices, long-term impact assessments, and collaborative efforts to address emerging challenges.

As the field of digital health continues to evolve, a sustained collective effort will be required to thoughtfully integrate these technologies into patient-centered care models. We hope the considerations presented in this paper will contribute to this ongoing dialogue. By using more systematic planning and development approaches, more effective next-generation DHTTs can be created, ultimately improving health outcomes and increasing health care system efficiency.

## Abbreviations

AI: artificial intelligence
COPD: chronic obstructive pulmonary disease
DHTT: digital health technology tool
FAIR: Findability, Accessibility, Interoperability, and Reusability
FDA: US Food and Drug Administration
FHIR: Fast Healthcare Interoperability Resource
GDPR: General Data Protection Regulation
HIPAA: Health Insurance Portability and Accountability Act
HITRUST: Health Information Trust Alliance
mHealth: mobile health
ML: machine learning
NIST: US National Institute of Standards and Technology
OECD: Organisation for Economic Co-operation and Development
RCT: randomized controlled trial
